# Frequency of single nucleotide polymorphisms in *NOD1 *gene of ulcerative colitis patients: a case-control study in the Indian population

**DOI:** 10.1186/1471-2350-10-82

**Published:** 2009-09-01

**Authors:** Ravi Verma, Vineet Ahuja, Jaishree Paul

**Affiliations:** 1 School of Life Sciences, Jawaharlal Nehru University, New Delhi 110067, India; 2 Department of Gastroenterology, All India Institute of Medical Sciences, New Delhi, India

## Abstract

**Background:**

Epidemiological studies have provided enough evidence that genetic factors have an important role in determining susceptibility to IBD. The most significant finding in the IBD research has been identification of mutations in the gene that encodes Nod2 (nucleotide-binding oligomerization domain 2) protein in a subgroup of patients with Crohn's disease. However, a very similar gene encoding Nod1 protein still has not been well documented for its association with Ulcerative colitis patients. Detection of polymorphism in *NOD1 *gene using SNP analysis has been attempted in the present study. We evaluated frequency and significance of mutations present in the nucleotide-binding domain (NBD) of *NOD1 *gene in context to Indian population.

**Methods:**

A total of 95 patients with ulcerative colitis and 102 controls enrolled in the Gastroenterology department of All India Institute of Medical Sciences, New Delhi were screened for SNPs by DHPLC and RFLP techniques. Exon 6 locus in the NBD domain of *NOD1 *gene was amplified and sequenced. Genotype and allele frequencies of the patients and controls were calculated by the Pearson's χ^2 ^test, Fisher's exact test and ANOVA with Bonferroni's correction using SPSS software version 12.

**Results:**

We have demonstrated DHPLC screening technique to show the presence of SNPs in Exon 6 locus of NBD domain of *NOD1 *gene. The DHPLC analysis has proven suitable for rapid detection of base pair changes. The data was validated by sequencing of clones and subsequently by RFLP analysis. Analyses of SNP data revealed 3 significant mutations (W219R, *p *= 0.002; L349P, *p *= 0.002 and L370R, *p *= 0.039) out of 5 in the Exon 6 locus of NBD domain of the gene that encompasses ATP and Mg^2+^binding sites. No significant association was observed within different sub phenotypes.

**Conclusion:**

We propose that the location of mutations in the Exon 6 spanning the ATP and Mg^2+ ^binding site of NBD in *NOD1 *gene may affect the process of oligomerization and subsequent function of the LRR domain. Further studies are been conducted at the protein level to prove this possibility.

## Background

Two diseases grouped under idiopathic bowel inflammation are ulcerative colitis (UC) and Crohn's disease (CD). Both diseases can be distinguished according to the differences in the clinical-pathological features [1]. The environmental factors and genetic predispositions participate in the emergence of the disease [2]. Several IBD linkage regions were identified in genome wide linkage scans [3]. Both CD and UC are considered complex genetic traits, as inheritance does not follow any simple Mendelian model [3]. The discovery of mutations in the *NOD2/CARD15 *gene (the first susceptibility gene known for CD associated mainly with an ileal involvement) in western European and north American countries as well as in Hungary was striking [4,5]. However, patient-control studies of Japanese, Chinese, Korean and Turkish populations did not encounter *NOD2/CARD15 *polymorphisms either in patients or control groups [6-10]. Nonsynonymous SNP scan for ulcerative colitis identified a previously unknown susceptibility locus at ECM1 and showed that several risk loci were common to ulcerative colitis and Crohn's disease (IL23R, IL12B, HLA, NKX2-3 and MST1), whereas autophagy genes ATG16L1 and IRGM along with *NOD2 *were specific for Crohn's disease [11].

Nod1 is a cytosolic protein and a member of a family of proteins known as the NLR/Nod (CATERPILLER family) [12]. Nod1 has been recognized as pattern-recognition receptor (PRR). *NOD1/CARD4 *and is located on chromosome 7p14 that has been genetically linked to asthma [13]. This protein family also includes a closely related protein Nod2. Both Nod1 and Nod2 are thought to function in inflammation, innate and adaptive immunity as well as in a variety of other processes that determine the balance between health and disease. These proteins are involved in recognition of intracellular bacteria primarily through sensing glycopeptides derived from microbial peptidoglycan. NLR family members are characterized as centrally located oligomerization and nucleotide-binding domain (NBD) that is followed by domain containing multiple leucine rich repeats at the carboxy terminal and caspase recruitment domain (CARD) at the amino terminal end [14]. However, Nod2 contains two CARD domain containing proteins [15]. Activation of Nod1 and Nod2 by ligand binding initiates a variety of cellular responses including Nf-kB and MAPK activation, cytokine production and apoptosis [16-18]. In the present study, we have attempted to study SNP in *NOD1 *gene in ulcerative colitis patients in order to determine if any significant mutation is associated with ulcerative colitis. The analysis is based on DHPLC technique which could screen accurately a large number of samples within a short period of time. The analysis was also validated by sequencing of PCR products and PCR-RFLP analysis.

## Methods

### Patients and Healthy controls

The study group consisted of 95 unrelated patients of Ulcerative colitis enrolled in the Gastroenterology department of All India Institute of Medical Sciences, New Delhi, India. 102 healthy controls matched for age and sex were also evaluated. The control subjects were healthy volunteers or patients with functional dyspepsia. They had no gastrointestinal or liver diseases. The diagnosis of UC was established according to clinical guidelines and criteria based on endoscopic, radiological, and histopathological examinations. The demographic and clinical features of Ulcerative colitis patients are represented in Table 1. Patients with UC were classified according to Montreal classification for age at onset, disease extent and behavior [19]. The mean age at diagnosis was 37 ± 12 years in UC patients and the mean disease duration was 4.3 ± 3.92 years. All patients and healthy controls gave informed consent and the study was approved by the ethical committee of the institute.

**Table 1:** Demographic and clinical features of UC patient.

	**UC (n = 95)**
Sex (M/F)	67/28

Duration of Disease, mean ± SD (range)	4.3 ± 3.92 (0.1-16)

Age at diagnosis (yr), mean ± SD	37 ± 12 (20-68)

15-40	66 (69.5%)

> 40	29 (30.5%)

Disease behaviour UC, *n *(%)	

Remission	18 (18.9%)

Mild	40 (42.1%)

Moderate	28 (29.5)

Severe	9 (9.5)

Disease extent UC, *n *(%)	

Rectum	46 (48.4%)

Left colon	27 (28.4%)

Pancolitis	22 (23.2%)

Smoking history	

Yes	8 (8.4%)

No	82 (86.3%)

Ex	5 (5.3)

Family history of IBD y/*n *(%)	1/94 (1.05%)
Appendectomy y/*n*, *n *(%)	4/91 (4.2%)

### DNA extraction

#### 1. Biopsy samples of patients

In the present study we have analyzed the genomic DNA from biopsy samples of patients since a parallel study is being carried out to study the gut bacteria profile during disease conditions. Genomic DNA was extracted from the colon biopsy samples (0.10 - 0.30 gm) according to the modified protocol of Taggart [20]. Tissue pieces were placed in 500 μl STE buffer (0.1 M NaCl, 0.05 M Tris-HCl and 0.01 M EDTA, pH 8.0), 10 μl SDS 10%, and 30 μl proteinase K (10 mg/ml). Solution was incubated at 50°C for 2 h for digestion. The tubes were inverted several times to accelerate the digestion process. After the digestion process was concluded, 3 μl of DNAse free RNAse was added, and incubated for 30 min at 37°C. The resulting digestion mixture was extracted once with 500 μl of buffer saturated phenol, pH 8, once with phenol-chloroform-isoamyl alcohol (24:1), and finally with chloroform-isoamyl alcohol. The DNA was then precipitated in 1 ml cold ethanol and sodium acetate (3 M, NaOAc, pH 5.3) (Maniatis *et al*., 1982). DNA pellets were dried and dissolved in 50 μl TE buffer (1 mM Tris-HCl, 7.6 and 0.1 mM EDTA, pH 8.0).

#### 2. Blood samples for controls

Genomic DNA was isolated for control individuals from peripheral blood leucocytes following standard protocols [21].

### Detection of polymorphisms

#### 1. DHPLC Analysis

Analysis of SNP was carried out using Denaturing High-Performance Liquid Chromatography on a fully automated WAVE DNA fragment analysis system equipped with a DNA Sep column (Transgenomic, Crewe, UK). Prior to DHPLC analysis, PCR products from a reference sample with known allele contribution were added in equimolar amounts to PCR products from all patient samples, denatured at decreasing temperature from 95 to 65°C to allow hetero duplex formation and the mixtures were automatically loaded onto the column with an autosampler. At a critical denaturing temperature, homo- and heteroduplexes were released off the column at different times. PCR products were examined for heteroduplexes by subjecting 20 μl of each PCR product to a denaturation step (10 min at 95°C) followed by gradual re-annealing step by decreasing sample temperature from 95 to 65°C over a period of 45 min. The PCR products were then separated (flow rate of 0.9 ml/min) through a 2% linear acetonitrile gradient and detected at 260 nm absorbance. The start concentrations of buffer B were selected by WaveMaker software version 4.1.40. (Transgenomic, Crewe, UK). The standard buffers were prepared from concentrated tri ethyl ammonium acetate (TEAA, 100 ml Transgenomic Part No. 553301) to give Buffer A = 0.1 M TEAA and Buffer B = 0.1 M TEAA plus 25% Acetonitrile. DNA was eluted with the following gradient consisting of buffer A (0.1 mol/L tri ethyl ammonium acetate) and buffer B (0.1 mol/L tri ethyl ammonium acetate containing 250 mL/L acetonitrile): 60% A-40% B for 30 s; 50% A-50% B for 5.5 min; 25% A-75% B for 10 s; 5% A-95% B for 1 min; and 60% A-40% B for 1.33 min. Wash buffer was 8% Acetonitrile. Analysis of each amplified sample took 8 min, including column regeneration and equilibration. The oven temperatures for optimal hetero duplex separation were determined using the WAVE maker software version 4.1.40 (Transgenomic, Crewe, UK), which gives a computer assisted determination of melting profile and analytical conditions for each fragment. The actual running temperature was established by repeatedly injecting the sample 1-2°C below and above the calculated temperature (64°C). Hetero duplex formation was checked by the melting profile of a known sequence of Exon 6. The temperature giving 70 to 80% double helical fraction of wild-type DNA was defined. Positive controls were used to determine the DHPLC conditions. A full list of primer sequence and annealing temperature for PCR amplification, resolution temperature and start concentrations of buffer B for DHPLC analysis are listed in Table 2. PCR fragments spanning the ATPase domain and Mg^2+ ^binding site were amplified from all 95 patients and 102 control individuals and screened further by DHPLC using the established gradient and temperature conditions.

**Table 2:** Primer sequences and DHPLC analysis conditions for two sets of designed primers.

**Exon**	**Primer sequence (5'-3')**	**Ampli. size(bp)**	**PCR anneal. temp.°C**	**DHPLC Oven temp.°C**	**Start % buffer B**
**N1Ex6-1**	F 5' AGCAGGTATACCCAGCAGCTGC 3' R 5' CCCGCTCTGGGTAGCAGTAGTG 3'	412	65.3	63.5	59-57
**N1Ex6-2**	F 5'CTACTGCTA CCAGAGCGGGA 3' R 5'CCGGAAGATGATCCAGCAGAA 3'	427	55	64.5	59-57

#### 2. Sequencing

The PCR products demonstrating differential DHPLC profile were subsequently cloned in pGEMT vector and sequenced on both strands to confirm the sequence variations. Sequencing reactions were performed with the ABI big Dye Terminator cycle sequencing kit v1.1 (Applied Biosystems, Foster City, CA, USA) and samples were sequenced on an ABI Prism 310 Genetic Analyzer (Applied Biosystems). The sequences were deposited to the NCBI database.

#### 3. RFLP analysis

In order to check the specificity of the DHPLC technique, the samples screened by DHPLC were further subjected to PCR-RFLP analysis. The PCR product of 840 bp (using N1Ex6-1forward and N1Ex6-2 reverse primer) was digested with *Eco88I *and *SdaI *to resolve mutations of E266K and L370R respectively. The PCR product of 427 bp (using N1Ex6-2 set of primers) was digested with *MbiI *to resolve mutations of 4773delG and PCR product of 412 bp (using N1Ex6-1 set of primers) was digested with *Eco81I *to resolve mutation of W219R. The restriction enzymes were procured from Fermentas. All the digestions were run overnight at 37°C, electrophoresed on a 2% agarose gel, visualized under UV illumination and stained with 0.4 mg/l ethidium bromide.

### Statistical Analysis

Data was evaluated by SPSS software version 12 using standard contingency χ^2 ^tests or Fisher's Exact Test for calculating Genotype frequency differences between cases and controls. A two-tailed P-value < .05 was considered significant. Hardy-Weinberg equilibrium was carried out using Pearson's chi square test to determine whether the proportion of each genotype obtained was in agreement with expected values as calculated from allele frequencies. Multiple comparisons were done using one way ANOVA based on the conservative Bonferroni correction. The significance level of α = .05 was chosen for all sets.

## Results

**Figure 1:**
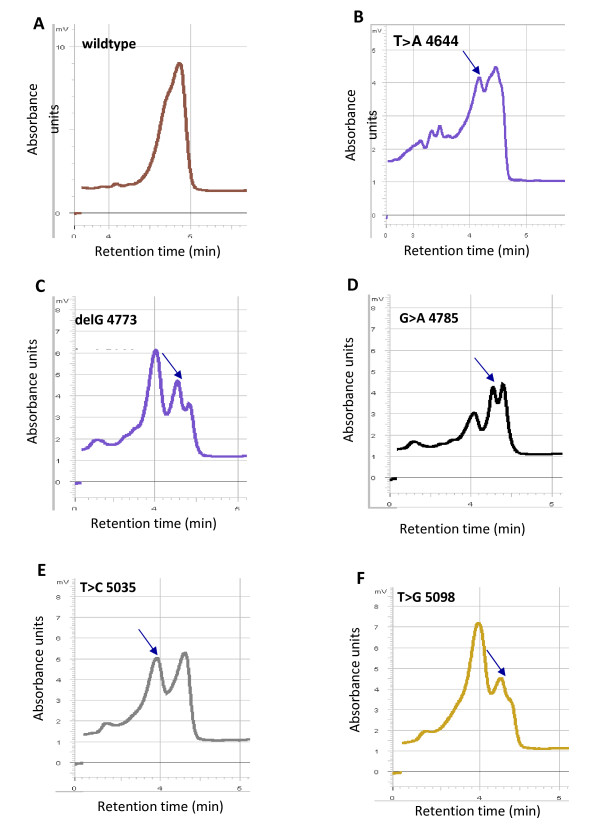
**Representative results of NOD1 mutation profile identified in Mg^2+ ^binding site and ATPase domain of Exon 6 by DHPLC analysis**.

Figure 1 represents the typical DHPLC chromatogram showing SNP profile of *NOD1 *gene in Exon 6 locus of NBD domain. The transitions observed in the ATP binding domain are represented in Figure 1B and Mg^2+ ^binding domain are shown in Figure 1C to 1F.

**Figure 2:**
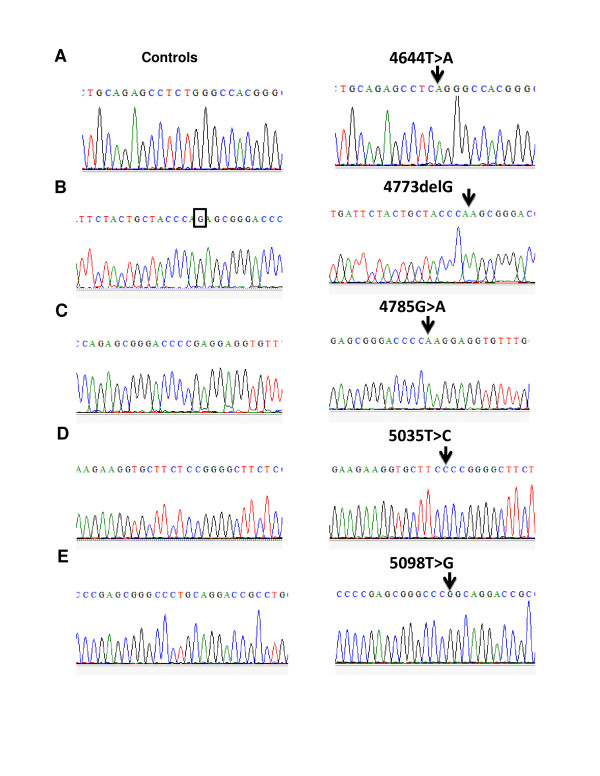
**DNA sequence electropherograms at exon6 locus from control and UC patients**. The nucleotide change is indicated by an arrow.

Homozygous nucleotide exchanges could be distinguished because of a slight shift in the elution time compared to the reference. The addition of an approximately equal amount of wild-type DNA to the sample (1:1) before the denaturation step allows homozygous alterations to be detected reliably. This step was taken for all the samples to identify homozygous sequence variations so that all the samples were analyzed first without mixing with an equal amount of wild-type DNA to detect heterozygous mutations. These were later confirmed by sequencing as shown in Figure 2. Table 3 represents the summary of SNPs in Exon 6 of *NOD1 *gene. Amino acid substitution from E266K was earlier observed by Walters et al, 2006 in CD patients [22].

**Table 3:** Summary of Mutations in Exon 6 of the *NOD1 *Gene

**Patient No.**	**mRNA mutation**	**Amino acid substitution**	**SubSNP (ss)**	**Predicted protein product**	**References**
33	4644T>A	W219R	ss104807147	Normal size	This report
4	4773delG	-		Aa261;PTC295	This report
16	4785G>A	E266K	ss104807137	Normal size	Walters et al., 2006
25	5035T>C	L349P	ss104807141	Normal size	This report
7	5098T>G	L370R	ss104807139	Normal size	This report

Nucleotide numbering is based on *NOD1 *gene available with GeneBank accession no. AF149774. The first base of the initiator methionine is taken as the start of the cDNA (PTC = premature termination codon at specified amino acid residue, aa = amino acid, E = Glutamic acid, K = Lysine, P = Proline, L = Leucine, R = Arginine, W = Tryptophan). ss represents the accession nos. of SNPs submitted to NCBI database.

**Figure 3:**
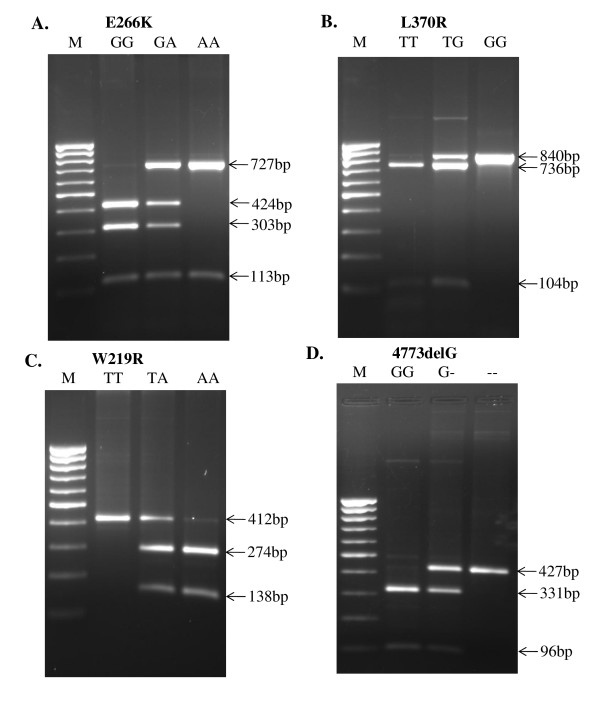
***NOD1 *E266K, L370R, W219R and 4773delG genotypes were deduced from the migration profile on a 1.5% agarose gel**. A. Wild-type DNA is visible as three bands 424 bp, 303 bp and 113 bp (GG), the mutated DNA is visible as double bands 727 bp and 113 bp (AA), where as heterozygotes show four bands (GA). B. Wild-type DNA visible as two bands 736 bp and 104 bp (TT), the mutated DNA is visible as a single band 840 bp (GG), where as heterozygotes exhibit three bands (TG). C. Wild type DNA is visible as a single band 412 bp (TT), the mutated DNA as double bands 274 bp and 138 bp (AA), whereas heterozygotes show three bands (TA). D. Wild-type DNA is visible as two bands 331 bp and 96 bp (GG), the deleted DNA is visible as a single band 427 bp (--), Heterozygotes are represented by three bands (G-). Lane M is a molecular weight marker of 100 bp (Fermentas).

RFLP analysis using selected restriction enzymes further confirmed the status of SNPs in our samples. Figure 3 shows representative results for genotyping of Exon 6 locus of *NOD1 *gene. To detect the nucleotide swap and to reconfirm our DHPLC data, RFLP was used. The variants were well distinguishable after restriction digestion (Figure 3). Transition of E266K (Figure 3A) could be detected in homozygous wild type when digested with *Eco88I *generating three bands 424 bp, 303 bp and 113 bp (GG), the mutated DNA was visible as a double band 727 bp and 113 bp (AA), whereas heterozygous type exhibited four bands (GA). Transition of L370R was studied after digesting the PCR product with *SdaI*. Wild-type (TT) yielded two bands of 736 bp and 104 bp, single band of 840 bp in homozygous mutated forms (GG), whereas heterozygous forms (TG) yielded three bands (Figure 3B). In W219R transition, three forms were resolved after digesting with *Eco81I*. Single band of 412 bp was observed in wild type (TT), two bands of 274 bp and 138 bp were observed in homozygous condition (AA) whereas three bands were observed as expected in heterozygous condition (TA) (Figure 3C). 4773delG mutation was detected after digesting the PCR product with *MbiI*. Two bands of 331 bp and 96 bp sizes were observed in wild type (GG), the deleted form was visible as a single band 427 bp due to missing of a nucleotide at the restriction site, where as heterozygous types yielded expected three bands (Figure 3D).

**Figure 4:**
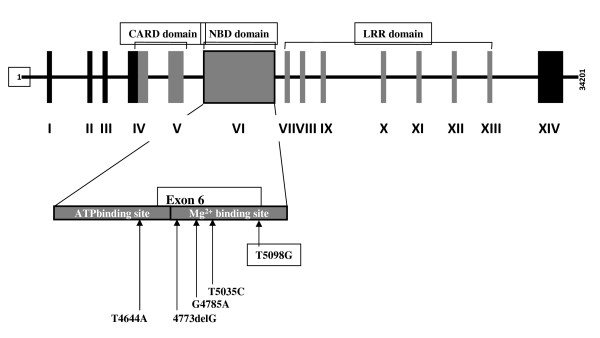
***NOD1 *gene with Exons and Introns**. Location of SNPs in Exon 6 are shown by arrows.

The genotypes and alleles distribution for *NOD1 *variants in UC and controls are compared at different loci of Exon 6 (Table 4). No significant departures were noted from the Hardy-Weinberg equilibrium (data not shown). Out of five SNPs reported in this study, frequencies of transitions fromW219R (*p *= 0.002); L349P (*p *= 0.002) and L370R (*p *= 0.039) were found to be significant whereas previously reported mutation of E266K did not show significant result in our study group. Figure 4 represents a comprehensive list of mutations detected and their location in the *NOD1 *gene. The nucleotide change T4644A leads to a change in the amino acid W219R that is located in the ATP binding site of the NBD domain whereas both L349P and L370R mutations are located in the Mg^2+ ^binding site of the domain.

**Table 4:** Genotypes and alleles distribution for *NOD1 *variants in UC and Controls

	**total**	**Wild-type**	**Heterozygous**	**Homozygous**	**Minor allele frequency**	**OR^1^**	**95% CI^1^**	***P*^2^**
						
						**UC vs. control**		
Patients								
4644T>A	W219R							
UC	95	61(64.2)	19(20.0)	15(15.8)	26			
Controls	102	89(87.3)	7(6.9)	6(5.9)	9	3.960	1.569-9.995	0.002*
4773delG								
UC	95	72(75.8)	15(15.8)	8(8.4)	16			
Controls	102	87(85.3)	10(9.8)	5(4.9)	10	1.853	0.900-3.812	0.091
4785G>A	E266K							
UC	95	53(55.8)	27(28.4)	15(15.8)	30			
Controls	102	68(66.7)	24(23.5)	10(9.8)	22	1.443	0.748-2.784	0.272
5035T>C	L349P							
UC	95	74(77.9)	12(12.6)	9(9.5)	16			
Controls	102	55(53.9)	29(28.4)	18(17.6)	32	0.308	0.144-0.656	0.002*
5098T>G	L370R							
UC	95	55(57.9)	19(20.0)	21(22.1)	32			
Controls	102	67(65.7)	24(23.5)	11(10.8)	23	2.326	1.033-5.238	0.039*

^1^ORs and probability values at 95% CI for disease status are shown.

^2^p-values were calculated with the Fisher's Exact test when comparing controls and UC patients.

*P *values refer to the UC vs. controls. *showing significant *P *value.

In order to establish any genotype/phenotype correlation, genotype and allele frequencies of the following SNPs were stratified by phenotypic sub groups. Analysis of the allele and genotype frequencies of 4644T>A, 5035T>C and 5098T>G in 95 UC cases at the Exon 6 locus showed no association with age, gender, smoking history, and appendectomy based on the Montreal classification (Table 5). More specifically in case of 4644T>A, we observed an increasing trend in the frequency of the A allele in disease activity being lowest in cases of remission (19.4%), 25% in moderate category, 26.2% in mild and 38.9% in severe category, however the differences did not reach statistical significance. Similarly, in case of 5035T>C SNP, we observed lowest frequency of disease extent in rectum (1.5%) that increased to 22% in case of pancolitis and 25% in case of left colon. The disease extent in case of 5098T>G being minimum (25.8%) in rectum followed by 32.1% in left colon and 38.2% in Pancolitis cases.

**Table 5:** Genotype and allele frequencies of 4644T>A, 5035T>C and 5098T>G SNPs in UC cases stratified by phenotypic subgroups, (n)

**SNP/gene**	**ss104807147 4644T>A**					**ss104807141 5035T>C**					**ss104807139 5098T>G**				
		
**genotype**	**TT (61)**	**TA (19)**	**AA (15)**	**Total (95)**	**Freq(A) 0.257**	**TT (74)**	**TC (12)**	**CC (9)**	**Total (95)**	**Freq(C) 0.157**	**TT (55)**	**TG (19)**	**GG (21)**	**Total (95)**	**Freq(G) 0.321**
**Sex**															
**Male**	40	16	11	67	0.283	52	10	5	67	0.149	35	15	17	67	0.365
**Female**	21	3	4	28	0.196	22	2	4	28	0.178	20	4	4	28	0.214
**Age at diagnosis (yr)**															
**15-40**	41	15	10	66	0.265	50	9	7	66	0.174	38	12	16	66	0.333
**> 40**	20	4	5	29	0.241	24	3	2	29	0.121	17	7	5	29	0.293
**Disease activity**															
**Remission**	13	3	2	18	0.194	13	3	2	18	0.194	13	2	3	18	0.222
**Mild**	25	9	6	40	0.262	33	3	4	40	0.138	22	10	8	40	0.325
**Moderate**	18	6	4	28	0.25	23	3	2	28	0.125	14	6	8	28	0.393
**Severe**	5	1	3	9	0.389	5	3	1	9	0.278	6	1	2	9	0.278
**Disease extent**															
**Rectum**	17	9	7	33	0.348	32	1	0	33	0.015	21	7	5	33	0.258
**Left colon**	18	4	6	28	0.286	19	4	5	28	0.25	16	6	6	28	0.321
**Pancolitis**	26	6	2	34	0.147	23	7	4	34	0.221	18	6	10	34	0.382
**Smoking history**															
**Yes**	4	4	0	8	0.25	6	0	2	8	0.25	5	2	1	8	0.25
**No**	54	13	15	82	0.262	63	12	7	82	0.159	46	17	19	82	0.335
**Ex**	3	2	0	5	0.2	5	0	0	5	0	4	0	1	5	0.2
**Appendectomy**															
**Yes**	2	1	1	4	0.375	4	0	0	4	0	2	1	1	4	0.375
**No**	59	18	14	91	0.252	70	12	9	91	0.165	53	18	20	91	0.319

## Discussion

This is the first report on the prevalence of the *NOD1 *polymorphisms in patients with Ulcerative colitis from northern part of India. The DHPLC scanning procedure described here has been found to be efficient and fast in screening and detecting point mutations in the samples including wild types as well as mutants. The sensitivity of the procedure was determined by sequencing the PCR products. We found this procedure well reproducible since the pattern of DHPLC chromatograms matched well with our expected sequencing data. This method has earlier been used for detecting point mutations for factor IX gene scanning [23] and other clinical applications [24]. We have successfully confirmed these results by RFLP analysis.

In the course of the study, DNA from biopsy samples as well as blood samples were analyzed since a parallel study is being carried out to investigate the importance of commensal bacterial flora and its communication strategies with the host during IBD.

We did not observe any difference between quality of DNA from colon biopsy samples versus blood. Several lines of evidence suggest that poorly regulated activation of the innate immune system could result in chronic inflammatory diseases. Mutations in domain NBD and LRRs of *NOD2 *gene are frequently observed in patients with Crohn's disease [7,25,26]. So far, association of *NOD1 *gene with ulcerative colitis patients has not been documented. We have chosen the Exon 6 spanning the NBD domain of *NOD1 *gene for our study. Earlier studies have shown that mutations within the exon encoding the nucleotide-binding domain in the CATERPILER gene family are associated with hereditary periodic fevers characterized by constitutive IL-1β production [27]. The CATERPILLER protein cryopyrin/NALP3 regulates IL-1β processing by assembling the multimeric inflammasome complex that is regulated by binding with ATP [27,28]. Mutation of the nucleotide-binding domain might affect ATP binding that may change the function of the following processes like caspase-1 activation, IL-1β production, cell death, macromolecular complex formation, self-association and association with the inflammasome component.

The mutation E266K in the *NOD1 *gene observed in the Exon 6 region was not significant (*P *= 0.272) in ulcerative colitis patients of Indian origin when compared with the non IBD population. However, this polymorphism has earlier been reported to be significantly associated with Crohn's disease susceptibility [5]. Although studied on a limited number of samples our data shows that this genotype does not demonstrate any association with ulcerative colitis. However new SNPs detected by us located in the Mg^2+ ^binding domain of the protein were L370R (*P *= 0.039) and L349P (*P *= 0.002). The third significant mutation was observed in the ATP binding domain of the gene, W219R (*P *= 0.002). These mutations are so far not reported in UC patients. The genomic organization demonstrates a high degree of conservation of the NBD- and LRR encoding exons and all the predicted NBD/LRR proteins are likely Mg^2+ ^and ATP binding proteins [29,30]. These domains play an important role in the oligomerization process thus any mutation in the ATP binding domain would lead to a defective oligomerization process due to non-availability of ATP required for this process. Deletion of G at 4773 position causing a frame-shift mutation observed in few Ulcerative colitis patients though not in a significant population, but can be predicted as a potential locus that give rise to a pre-termination codon at 295 position of the amino acid encoding a truncated protein that may affect the function of *NOD1 *gene considerably. Interestingly, we observed and recorded that the patients showing this variant exhibited symptoms of acute inflammation.

We have not found any significant association between the different genotypes and the demographic data on the patients or the clinical characteristics of UC though there was an increasing trend in frequency of 5035T>C variant allele in the disease extent from rectum to pancolitis and left colon without significant association with any sub-phenotypes.

Certain limitations of our data like limited size of the samples must be considered when we are interpreting our data. Before, we can make a firm conclusion on association of these mutations with the disease, there is a need for replication in an independent cohort. Given the importance of these results, further confirmatory studies are warranted in larger UC population.

## Conclusion

Screening of samples for SNP analysis using DHPLC technique has been quite useful and less time consuming in analyzing large number of patients samples. This high-throughput genotyping technique is particularly suitable for routine diagnosis of SNPs.

Our study confirms association of three SNPs to ulcerative colitis. Significant mutations observed in ATP (W219R, *p *= 0.002) and Mg^2+ ^(L370R, *p *= 0.039 and L349P, *p *= 0.002) binding domains of Exon 6 may lead to a defective oligomerization of protein which subsequently may lead to a 'loss of function' by preventing the recognition of MDP that is necessary for subsequent NF-kB activation

## Abbreviations

(NOD1): Nuceotide oligomerization domain; (UC): Ulcerative Colitis; (NBD): Nucleotide Binding domain; (IBD): Inflammatory Bowel Disease; (SNP): Single Nucleotide Polymorphism.

## Competing interests

The authors declare that they have no competing interests.

## Authors' contributions

JP conceived and coordinated the study. RV carried out the genotyping experiments. JP and RV drafted the manuscript and conducted the statistical analysis. VA made the diagnosis of the patients and collaborated in collection of the samples. All authors read and approved the final version.

## Pre-publication history

The pre-publication history for this paper can be accessed here:


